# Use of Diagnostic Imaging Modalities in Modern Screening, Diagnostics and Management of Breast Tumours 1st Central-Eastern European Professional Consensus Statement on Breast Cancer

**DOI:** 10.3389/pore.2022.1610382

**Published:** 2022-06-08

**Authors:** Gábor Forrai, Eszter Kovács, Éva Ambrózay, Miklós Barta, Katalin Borbély, Zsolt Lengyel, Katalin Ormándi, Zoltán Péntek, Tasnádi Tünde, Éva Sebő

**Affiliations:** ^1^ GÉ-RAD Kft., Budapest, Hungary; ^2^ Duna Medical Center, Budapest, Hungary; ^3^ Mamma Egészségügyi Zrt., Budapest, Hungary; ^4^ Royal Cornwall Hospital, Truro, United Kingdom; ^5^ National Institute of Oncology, Budapest, Hungary; ^6^ Ministry of Human Capacities, Budapest, Hungary; ^7^ Hamad Medical Corporation, Doha, Qatar; ^8^ University of Szeged, Szeged, Hungary; ^9^ Dr Réthy Pál Member Hospital of Békés County Central Hospital, Békéscsaba, Hungary; ^10^ Kenézy Gyula University Hospital, University of Debrecen, Debrecen, Hungary

**Keywords:** mammography, breast ultrasound, breast MRI, breast screening, conventional nuclear medicine, SPECT/CT, PET/CT, biopsy

## Abstract

Breast radiologists and nuclear medicine specialists updated their previous recommendation/guidance at the 4th Hungarian Breast Cancer Consensus Conference in Kecskemét. A recommendation is hereby made that breast tumours should be screened, diagnosed and treated according to these guidelines. These professional guidelines include the latest technical developments and research findings, including the role of imaging methods in therapy and follow-up. It includes details on domestic development proposals and also addresses related areas (forensic medicine, media, regulations, reimbursement). The entire material has been agreed with the related medical disciplines.

## Introduction

Radiologists and nuclear medicine specialists specializing in the diagnostics of breast diseases have compiled their opinions on diagnostic imaging and screening for breast cancer. Based on international evidence, it is hereby recommended that the radiological and nuclear medicine aspects of breast cancer diagnosis and management are conducted in accordance with these guidelines. This material was discussed and accepted by the 4th Breast Cancer Consensus Conference on 28–29 August 2020. It was then submitted to the Radiology Section of the National Advisory Board, which has approved it. Regular updates of the material are still recommended.

## Purposes of Diagnostic Imaging Methods in Breast Tumours

• Breast tumour screening, detection, confirmation (1).
• Guiding targeted biopsy: preoperative/pre-therapeutic sampling to establish cytological/histological diagnosis, whenever requested.
• Assessment of locoregional extent.
• As part of therapeutic planning, staging.
• As part of therapy: preoperative localization of the tumor bed or tumor site with markers effective neoadjuvant therapy, confirmation of a tumour in the specimen, helping with pathological processing, percutaneous minimally invasive therapy in selected cases.
• Evaluation of therapy effectiveness.
• Follow-up studies.
• Early detection of recurrence.
• Participation in new staging.

The basic principle: No breast therapy may be performed without imaging studies.

## Breast Investigation Modalities

### Mammography

Mammography is mandatory for symptoms or complaints developing in patients aged over 30–35 years. In justified cases, it can be carried out in patients aged under 30. Mammography is the only scientifically proven method for screening asymptomatic women at average risk with the purpose of reducing breast cancer mortality (2). Direct digital mammography has been shown to perform better than conventional analogue techniques (3). As the screening age for mammography varies from country to country, the age cut-off for mammography and US scans should be adjusted accordingly.

### Tomosynthesis

Digital breast tomosynthesis (DBT) is a procedure based on full-field digital mammography (FFDM) in which an X-ray tube moving in an arc capturing 10–15 overlapping digital images of the breast in a short time at low radiation doses. Data are computer- processed, resulting in thin slice images and can be reconstructed to summation images called “synthetic 2D images,” which look similar to conventional images. In order to reduce radiation dose, it is recommended that conventional 2D images be partially or completely replaced with synthetic 2D images, provided that the device has an official certificate (e.g., FDA approval). 3D tomosynthesis is more sensitive for the assessment of breast structure, and hidden lesions are easier to be detected (higher sensitivity). Tomosynthesis is highly efficient (higher specificity) in assessing overlapping tissues (summation) that pose diagnostic difficulties during conventional 2D imaging. By analysing images cut into thin slices, breast structure can be assessed without the disturbing effects of overlapping, so that pathological structural distortions and lesion borders can be evaluated more accurately, and false-positive results resulting from summation can be eliminated. As a result, 29%–41% more tumours can be detected and, if applied during screening, recall rates are significantly reduced, and unnecessary biopsies can be avoided. Use of tomosynthesis in breast screening is particularly advantageous for breast structures (dense fibrotic, fibroadenotic tissue) for which conventional mammography has a lower sensitivity (4, 5).

### Contrast-Enhanced Spectral Mammography

One of the latest developments in digital mammography is the use of intravenous iodinated contrast media for dual-energy mammography. The subtraction technique allows for analysis of contrast accumulation in breast lesions, similarly to breast MRI. According to some studies, CESM may be suitable for the assessment of abnormalities detected by mammography, especially for dense breast structures, to evaluate the extent of the disease. According to some reports, its sensitivity is close to that of a breast MRI, but this has not yet been clearly established for DCIS. Radiation exposure is 81% higher than for a conventional 2D digital mammography, and 48% higher than for DBT (6–8). Currently, this modality is being researched and may only be used with serious reservations, and it must never be a substitute for indications that have long been supported by evidence (e.g., mammography, breast MRI) (9, 10).

### Ultrasound Scanning of the Breast

Breast ultrasound can be used on its own in patients aged under 30. Over the age of 30–35 years, it can be a complementary procedure to mammography, when needed (11). It is not suitable for breast cancer screening, at any age. As for ultrasound scans of other regions, breast ultrasound scanning should be documented with images in accordance with professional rules, even in negative cases. Colour Doppler is optional, but can be used in addition. Some studies suggest that a significant number of malignancies can be detected by ultrasound scanning as a complement for mammography (12), but this has not yet been routinely introduced due to extra human resource requirements and a high false positive rate.

### Automated Breast Ultrasound

Automated breast ultrasound scan hasn’t become widespread yet as a complementary investigation modality for dense breast structures (13, 14). Using a probe covering the breast, volumetric data are collected about the entire breast, from which slices can be reconstructed to review the glandular tissue in the main anatomical planes. This modality provides a good anatomical overview, as it is reproducible, and it can be complemented by an automatic image recognition system. Its disadvantage is that the false positivity rate is high for the biopsies it indicates, most of which will be benign (15). It should be emphasized that the resolution and information content of ultrasound images provided by ABUS is the same as for manual ultrasound scanning.

In most states of the United States, for high density breasts, it is mandatory to inform patients about investigations that complement screening mammography (e.g., ultrasound). Mammographic breast density, as an independent risk factor, is still subject to scientific debate; however, the tumour-masking effect of a higher density, which makes tumour detection difficult, is an accepted fact (16).

### Hybrid—DBT and ABUS

This is a combination of digital breast tomosynthesis and automated breast ultrasound. This modality is part of a research project, and it is not yet commercially available. This device captures tomosynthetic mammography images in a conventional CC and MLO setup, after which 3D ultrasound images are recorded by an automatic ultrasound device built into the compression plate. Studies have shown that the combination of ultrasound scanning and mammography in screening may significantly improve the rate of detected abnormalities. This method utilizes the advantages of both tomosynthesis and automated ultrasound scanning over the 2D technique (17, 18).

### Second-Look (Repeated Targeted) Ultrasound

If an MRI image suggests malignancy, targeted (second look) ultrasound scanning is recommended even if the lesion was hidden on mammography and on the first ultrasound scan. It is important that this is done by a radiologist experienced in breast MRI. By doing this, 60%70% of originally occult lesions can be detected, and ultrasound-guided sampling can be performed (19).

### Elastography

Shearwave sonoelastography is a non-invasive imaging procedure based on tissue elasticity, measured in kPA. An abnormal process will modify the elastic properties of the affected tissue (20). According to studies, ultrasound elastography may help differentiate BI-RADS 3 and 4a lesions, and may increase the specificity of ultrasound scanning, thereby reducing the number of unnecessary breast biopsies (21, 22). The role of elastography in the monitoring of neoadjuvant treatments, in the differential diagnosis of suspected axillary lymph nodes, and in the evaluation of microcalcifications affecting the glandular tissue has been investigated. This method has also been integrated into the current BI-RADS lexicon of 2013 (23).

### Breast MRI

#### Indications for Breast MRI

• If a tumour is suspected, but the results of mammography and ultrasound are insufficient or uncertain (24).
• When searching for an occult primary tumour.
• Preoperative assessment of proven cancers, for the evaluation of multiplicity, extent, bilaterality, chest wall involvement—especially if different investigation methods show a difference in size (difference of more than 1 cm between mammography and ultrasound, especially in patients aged under 60).
• Breast MRI has been shown to be of outstanding importance in assessing the extent of an invasive lobular carcinoma (preoperative MRI changes therapy by 28% and significantly reduces the number of reoperations) (25).
• Preoperative MRI is also a useful method for the assessment of DCIS/EIC extent.
• If multifocality is suspected on MRI, efforts should be taken to confirm this histologically; if it cannot be confirmed, the original breast-conserving surgical plan may be overridden by mastectomy only by an oncological team decision or by the patient’s wish.
• To increase sensitivity in the screening of dense breasts.
• To differentiate recurrence/scar/granuloma/fat necrosis (not always differentiable without biopsy).
• Screening in high-risk patients (26).
• For planning and monitoring the effects of neoadjuvant treatment (27).
• For planning partial breast irradiation (PBI).
• To examine the integrity of a breast implant, to look for implant rupture (especially if physical signs are present), if the result of this examination will influence the treatment.

Important note: In premenopause, contrast-enhanced breast MRI should be performed at week 2 or possibly week 3 of the cycle, otherwise the false positive rate will be very high.

#### Contraindications for Breast MRI

• General contraindications for MRI (e.g., pacemaker, etc.)
• Nonspecific clinical symptoms (e.g., breast pain) with negative mammography and ultrasonography results.
• MRI should not be used instead of biopsy for lesions that can be evaluated only pathologically, e.g., to characterize microcalcification.

#### Relative Contraindications for Breast MRI

• Due to a limited evaluability, it is generally not recommended for 6 months after surgery and within 12–18 months after radiation therapy, except for special cases (and only after prior consultation with a radiologist).
• After a core/vacuum-assisted biopsy, there is no need to wait before MRI scanning, but if possible, it is recommended that it should be delayed for a couple of weeks: it is advisable to wait for any haematoma to be absorbed, although this does not usually interfere with diagnosis.
• Metal clips inserted during surgical or radiological intervention do not interfere with breast MRI; however, the filling valve of some expander implants may make scanning impossible due to their ferromagnetic material.
• Pregnancy (see below).

Important note: By default, MRI is not required for implanted breasts for either screening or diagnostic purposes.

#### Breast MRI Is Not Indicated

• For histological characterization in cases where a targeted biopsy can be performed (differentiation of scar site recurrence, for characterizing microcalcifications, nodules of unknown nature, etc.)
• In the event of uncertain cytological examination with non-informative (C1) or borderline (C3) results (in such cases a core biopsy should be performed).
• For the accurate evaluation of axillary lymph nodes.
• Instead of mammography, if the patient has radiophobia.
• For routine follow-up of operated, treated patients instead of mammography or ultrasound.

#### Promising Breast MRI Indications Still Under Investigation

• Examination of discharging breasts and to support therapeutic decision-making for B3 lesions (24).
• A large multi-centre study (Preoperative Breast MRI in Clinical Practice: Multicenter International Prospective Meta-Analysis [MIPA] of Individual Data) is ongoing to demonstrate that a breast MRI scan would be required before treating any confirmed tumour. Several studies have found that preoperative MRI modifies therapy by 15%–25%, but their statistical power is not yet sufficient to make this recommendation general (25).
• MRI spectroscopy is still in the research phase. This special procedure may increase the specificity of assays by detecting a tumour-specific component (e.g., a choline peak).

### Ductography (Galactography)

Ductography may be used when an intraductal process is clinically suspected if this cannot be excluded by other imaging and intervention methods. It can also be used for dye marking of affected ducts before surgery. Because of its low sensitivity and specificity, it is not suitable for excluding an intraductal process in the event of a negative result. In some countries (e.g., the United Kingdom) it has been removed from the list of interventions used in practice. MRI has started to take over the role of ductography. Based on a large review study, the sensitivity and specificity of MRI (92% and 97%, respectively) for carcinomas are significantly higher than for galactography in the diagnosis of patients with discharging breasts. In the event of negative mammography and ultrasound results, MRI scanning is recommended as a next step of assessment (28).

### F-Fluorodeoxyglucose Positron Emission Tomography/Computed Tomography

• Not suitable for breast screening (29–38).
• If breast cancer is suspected, routine testing is not justified because of its low sensitivity in the detection of tumours that are
  ○ Less than 5 mm in diameter and
  ○ Those with low FDG avidity (DCIS, LCIS, low-grade lobular carcinoma, tubular carcinoma)
• PET/CT is less suitable than breast MRI for searching for occult breast tumour.
• 18F-NaF PET/CT may be chosen as an alternative to conventional bone scintigraphy (not yet reimbursed in Hungary).

### Positron Emission Mammography

Positron emission mammography alone is not suitable for breast screening. PEM is a dedicated breast camera with a resolution of 1–2 mm that can be used as a complementary method to mammography and breast ultrasound. It is primarily recommended in patients in whom MRI scanning is not indicated or not feasible for any reason. Its sensitivity and specificity in the identification of malignant foci within the breast are nearly identical to those of MRI. It can be used to determine multiplicity within the breast, to differentiate scar and tumour in an operated breast, and to measure response to chemotherapy. Stereotactic sampling systems used in mammography can also be used for PEM (device dependent). When using the method, radiation exposure (3.0–3.5 mSv) to the radiopharmaceutical used (which is not focused to the breast) should be taken into account (39). Not available in Hungary.

### Positron Emission Tomography/Magnetic Resonance Imaging

PET/MRI is a promising technique that is still primarily used for research; its use is recommended in patients for whom PET and MRI indications coexist and minimization of radiation exposure is essential (40, 41).

## Interventional Procedures

The result of preoperative/pre-therapeutic complex diagnostics should provide sufficient certainty for the operating surgeon to plan the surgery accurately and/or for the oncologist to choose the therapy.

In the event of a positive (malignant) aspiration cytology (FNA) result, a consensus must be reached between the pathologist, oncologist, surgeon, radiologist and the patient when establishing the indication for surgery/therapy, along with a correlation between the radiological and pathological results.

Breast screening and diagnostic study sites should provide the opportunity (or a background in another facility) for guided sampling for all imaging procedures (mammography, ultrasonography). (MRI-guided intervention is currently not available in Hungary.) For an image-guided intervention, it should be documented through images that the device has reached the lesion and sampling conditions (target description, exact location [quadrant/clock face/distance from nipples/fold], device, targeting, validation, clip position) must be recorded.

Efforts should be taken to obtain a definitive diagnosis from the first sampling, and there should in any case be no more than two samplings. To do this, an appropriate sampling and guiding type should be chosen.

### Guiding Biopsy

Sampling should always be guided by an imaging technique, for both palpable and non-palpable lesions.

• Ultrasound-guided sampling of the breast and regional lymph nodes is recommended if the palpable or non-palpable lesion is clearly visible on ultrasound.

It is strongly contraindicated that after an ultrasound-guided sampling with a benign result. Lesions which are not well identifiable by ultrasound are followed-up only.

• Mammography-guided (stereotactic) sampling is required for non-palpable, non-ultrasound-identifiable lesions that are not certainly benign, e.g., microcalcifications. Aiming can be done in a sitting/lying/or side lying position. Lesions visible only on tomosynthesis (mostly structural distortions) may only be aimed at by tomosynthetic stereotaxis (which cannot be replaced by MRI). The latter method is not currently funded by the NEAK (National Health Insurance Fund of Hungary).
• MRI-guided sampling (42) is performed when a uncertain or suspicious lesion detected by contrast enhanced MRI, not visualized by mammography or ultrasound, a decision cannot be made as to whether the lesion is benign or malignant. Sampling should be performed in a vacuum-assisted manner, and a marker clip should be inserted after the procedure.

### Biopsy Tools—Aspiration Cytology, Core Biopsy, Vacuum-Assisted Biopsy

Fine needle aspiration cytology (FNA, FNAB, FNAC), core biopsy, and VAB are all extremely important in diagnosis and therapeutic planning. Cytology is a faster, cheaper, but more inaccurate procedure (more false negatives and non-evaluable specimens), while core biopsy is more accurate (histological type, immunohistochemical parameters, definitive confirmation of benignity), and usually eliminates errors in evaluating fibrotic lesions and lesions in treated breasts. Because of the low reliability of FNA, it is contraindicated in some countries for breast diagnostics—except for evaluation of fluid-containing structures.

In some cases, VAB is the first choice of method according to current recommendations.

Detailed, state-of-the-art professional recommendations and possibly local availability should be considered when choosing a biopsy procedure (device/needle), except for the following cases:

• Vacuum-assisted biopsy (VAB) (43) is the gold standard for evaluating microcalcifications, but in selected cases (lesions larger than 10 mm, etc.) a conventional core biopsy may be sufficient. FNA is not suitable for the diagnosis of calcifications, partly since the effectiveness of sampling (presence of calcification in the sample) cannot be validated.
• In order to validate the biopsy of calcifications, specimen mammography of the tissue cylinder is mandatory; the presence of calcifications must be stated in the biopsy report. If calcification cannot be visualized within the tissue cylinder on specimen mammography, sampling (in the event of a negative result) cannot be considered representative, and therefore a therapeutic or follow-up decision cannot be made based on this.
• If FNAB is performed in an atypical lesion or a lesion suspected of malignancy (RKU 3, 4, 5, BI-RADS 4, 5), a negative or benign cytology result cannot be accepted to rule out malignancy, when a benign lesion diagnosed on FNA (C2) is not clearly stated or if the radiopathological correlation is questionable or it is not seen.
• If for any lesion, proper information cannot be obtained for therapeutic decision-making using repeated, adequate sampling with a higher-level biopsy method, surgical excision may be required.
• Core biopsy should be performed in all cases when it is requested for therapeutic planning or by protocols of other disciplines (surgery, oncology) (e.g., for neoadjuvant treatment, mastectomy, axillary dissection).
• Default needle size for core biopsy: 14G. In case of suspected carcinoma insitu/microcalcifications, the use of a needle sized 12G is recommended; default needle size for vacuum-assisted biopsy: 7G–9G.
• None of the sampling procedures is suitable for definitive diagnosis in papillary lesions, ADH and some other B3/C3 cases, which require surgical or vacuum-assisted excision and complete histological processing. In the event of an insitu carcinoma, none of the sampling methods is suitable to rule out a possible invasion.
• For cytology from a lesion in any tissue type (except lymph nodes) that fails or has uncertain results, core biopsy is usually required, and not repeat cytology.
• In the event of a failed core biopsy (for non-technical reasons), vacuum-assisted sampling should be considered instead of a repeated core biopsy.
• If the need for sampling has already been stated for a lesion (i.e., the suspicion of malignancy has arisen with any probability), follow-up cannot be recommended without establishing a specific diagnosis (e.g., for a C1 result).
• During preoperative diagnostics, an abnormal radiological lesion may be completely removed, and in such cases placement of a marker clip is imperative.

## Algorithms for Assessment

### Screening for Breast Cancer

Organized public health screening: a nationally organized invitation-based screening programme for women with a medium risk aged 45–65, every 2 years in Hungary (other countries: see Table 1) (1, 2, 44–52). (A public health programme initiated by the health care system as a provider, publicly funded or involving population groups considered to be at risk, implemented with a professionally justified frequency.) (53).

**TABLE 1:** Timelines of breast-screening programmes with age covered in studied countries by Central and Eastern European Academy of Oncology as reported by panel members.

Country	Implementation of screening programmes	Age covered
Armenia	Pilot 2021–2023 in 3 of 11 regions of the country	50–69
Azerbaijan	2008	30–70
Bulgaria	2012	45–69
Georgia	2008	40–70
Hungary	2001–2002	45–65 (soon will be modified to 40–75)
Kazakhstan	2008	40–70
Poland	2006	50–69
Russian Federation	2006	40–75
Romania	2008	50–69
Serbia	2012/13	50–69
Slovakia	2019	50–69

Individual (opportunistic) screening: occasional imaging studies of women over the age of 40 years at average risk, with no symptoms suggestive of tumour, no history of breast cancer, for ruling out breast cancer. (Occasional use of methods suitable for recognizing a hidden target condition, related to other medical activities or spontaneously required.)

Assessment methods:

• Physical examination + mammography (medical technician).
• Evaluation of mammography: double medical reading (radiologist).
• In case of positive or doubtful results, the patient should be recalled for a complex diagnostic breast assessment (additional investigations), which is needed to clarify the issue: targeted, zoomed, etc. images, ultrasound scanning, guided sampling, MRI, etc.

Screening of high-risk women (26, 54–59): mutations in the currently known “breast cancer genes” explain 25%–30% of familial breast cancers; other predisposing genes are still unknown. Detection of missing genetic heritability is a central theme of current research (60, 61). Based on this knowledge, it is considered important that in cases of confirmed familial breast or ovarian cancer BRCA1,2 mutation, Li–Fraumeni syndrome, Bannayan–Riley–Ruvalcaba syndrome, Cowden syndrome, Peutz–Jeghers syndrome, and a history of chest radiation administered 10–30 years previously, screening recommendations should also apply to individuals with a breast cancer risk above 20%–25% according to validated mathematical tests. Among mathematical models, the best known are: BRCAPRO, BOADICEA, modified BOADICEA, (2008) Gail, Claus, Tyrer-Cuzick, Myriad I/II and COUCH models. It is advisable to use models that also take into account an extended family history. It should be noted that the National Institute of Health and Care Excellence (NICE) in the UK recommends the use of BOADICEA to decide whether to carry out MRI screening of high-risk patients (62).

Screening recommendation in Hungary for the high-risk group: Above the age of 30 years, mammography (2D digital mammography, or possibly with 3D tomosynthesis and 2D synthetic software) and ultrasound scanning, complemented by annual MRI (when possible), is recommended: at least from the age of 30 years for known BRCA1/2 carriers, and at least from the age of 20 years for those with TP53 mutations (35). Omitting use of mammography screening has to be considered at patients with Li-Fraumeni syndrome, due to the risk of secondary radio-induced malignancies (63).

Hormonal induction (*in vitro* fertilization programme): Most data in the literature do not support an increased risk of breast cancer after fertility-enhancing hormone treatments, although there is always a theoretical chance of this occurring. Based on individual judgement (mainly after repeated or long-term treatments), annual mammography screening should be considered in women undergoing such treatment (64).

### Diagnostic (Clinical, Complex) Breast Assessment

Detailed assessment and individualized screening of patients who have complaints, and of those revealed by screening. The purpose is to establish a maximally accurate preoperative/pre-therapeutic (non-operative) diagnosis (preferably complemented by cytology/histology sampling) in order to optimize the malignant/benign ratio for cases undergoing surgery. According to EU protocol indicators, at least 90% of cases of confirmed malignancy require a preoperative biopsy at the time of diagnosis (35, 65, 66).

In terms of workforce, the recommendation is that all steps of complex breast diagnostics be performed by either one radiologist or as few radiologists as possible, so the diagnosis, based on information provided by each modality and interventions will be as accurate as possible.

Referral to mammography/ultrasonography: since the choice and feasibility of imaging methods required for an individual patient depend on several factors (clinical questions, age, breast size, etc.), it is recommended that the patient be referred for “complex breast assessment” instead of “mammography” and “breast ultrasound”, and the investigating physician should decide what investigations they consider necessary, depending on the clinical question.

Above the age of 30–35 years (age limit should be determined on an individual basis and is the competence of the radiologist):

• Physical examination (physician or certified nurse) + mammography (medical technician).
• Evaluation of mammography: single medical reading (radiologist).
• Additional ultrasound scanning (evaluation by the radiologist): palpable, circumscribed lesion, hyperdensity, discharging, inflammatory, operated, implanted, non-involutional, dense breasts with a complex mammographic structure, in cases of high risk, etc. (67).
• Sampling, if necessary.
• It is recommended that a breast MRI be performed if mammography, ultrasound and sampling did not provide enough information, but only when confirmation of diagnosis by MRI can be expected (and only based on a preliminary radiological consultation).

Under the age of 30–35 years (age limit should be determined on an individual basis and is the competence of the radiologist):

• Physical examination (physician or certified nurse) and ultrasound scanning (67).
• Evaluation of ultrasound scan: single medical reading (radiologist).
• Mammography, if needed (women who have given birth, for large breasts, in high-risk cases, in individual cases, etc.) with a single reading (radiologist).
• Sampling, MRI, etc., if needed: see the previous paragraph.

### Follow-Up of Lesions

Follow-up over time is sufficient only for lesions with a radiomorphology showing a probability of malignancy of less than 2% (BI-RADS category 2 or 3, or stable condition documented for at least 3 years, for solid lesions). If the probability is 2% or more and in the absence of a follow-up history, sampling is mandatory (23). Depending on the type of lesion, follow-up is usually performed in 6-month cycles, for up to 3 years. For inflammatory processes, follow-up in shorter cycles may be justified.

## Artificial Intelligence in Breast Diagnostics and Screening

The task of artificial intelligence (AI) is to implement human intelligence using computational models. The goal is to make computers capable of performing tasks that can be accomplished by human intelligence. Artificial intelligence is a system that displays intelligent behaviour, analyses its environment, and is able to act with a certain degree of autonomy to achieve a specific goal (68). Artificial intelligence is based on machine learning rather than on conventional computer programming. During this process, the computer is provided with a set of data and expected responses, after which the machine will create the rules. Based on the established rules, the machine itself will provide the answers based on new data. This also means that during the learning phase, it is worth using as much data as possible and that such systems are capable of continuous improvement. Radiology finds itself in a special situation also because, owing to digital image archiving systems that became widespread years ago, a huge database is now available, constituting a basis for such developments.

The CAD (computer-aided detection) systems used in the early 2000s were based on conventional programming. After initially promising results, these systems did not become widespread in everyday practice. The performance of the film reader radiologist did not improve, the number of recalls increased, but the rate of tumour detection did not improve (69, 70).

Artificial intelligence based on machine learning seems to be a promising development, with many studies showing encouraging results in reading mammograms captured on various devices, and many results show accuracy similar to human performance under research conditions (71–73).

Assessing breast density is important in many ways (diagnostic difficulty, medico-legal problems, individual risk). As a best practice, description of breast density in radiology reports is increasingly frequent; however, evaluation of this feature shows significant inter-observer differences. There are currently multiple breast density analysis systems on the market that have been approved by the FDA (74–76).

With the spread of digital tomosynthesis, the amount of information and time required for reading continues to grow, which further increases the need to find new solutions. Evaluation of image material generated during automated ultrasound scanning is another direction of development. Breast MRI scans have also attracted the interest of artificial intelligence development groups and companies (77). Evaluating the response to neoadjuvant treatment seems to be a promising area within this. Development of decision-making algorithms is also expected to receive a boost.

Currently, only recommendations based on limited evidence can be formulated. It is difficult to compare different studies, and a standardized method for comparison of studies and efficacy has not yet been established. At present, solutions based on artificial intelligence are not yet applicable in daily routine patient care (78). Results are expected in the following applications (79, 80):

• Assessment of breast density, individualized risk assessment.
• A combination of a radiologist and AI instead of a double reading.
• Highly reliable negative mammography reading by AI (without human intervention).
• Other imaging techniques and AI.
• Clinical decision support systems.

## Assessment Protocols

### Assessment of a Discharging Breast

• Physical examination—to be documented: colour, side, amount of discharge, whether it is spontaneous or appears on compression, number or possible localization of discharging ducts, duration (onset, continuous or intermittent, nature of change), other symptoms (e.g., inflammation) and whether the discharge is pathological (28).
• Non-pathological discharge: unilateral/bilateral discharge from multiple ducts.
  ○Actions: mammography (over 30–35 years) and ultrasound (under 30–35 years only ultrasound), discharge (contact) cytology (at onset).
  ○ If these investigations have negative results, no further diagnostic actions are needed.
• Pathological discharge: bloody, serous or colourless discharge from one duct (especially if unilateral), usually spontaneous and persistent.
  ○ Actions: mammography (over 30–35 years), ultrasound, discharge cytology.
  ○ In 35%–56% of cases, this is caused by papilloma or duct ectasia, and by DCIS or IDC in 5%–23% of cases. If image is suggestive of intraductal papillary lesion or DCIS, IDC, assessment should be continued according to guidelines for solid structures or malignancy.
• If mammography and ultrasound scanning show negative results and blood or other signs of epithelial proliferation are found in the discharge on cytology examination, MRI or galactography may clarify the cause of the discharge, and location, multiplicity, and extent of underlying lesion(s). Of the two modalities, MRI is preferred because of its higher sensitivity and higher specificity.
• If either method yields positive results, it is recommended that ultrasound scanning be repeated and mammography re-evaluated, or possibly that additional images be captured to reveal the lesion. If a lesion is identified, a core biopsy is required.
• If the clinical picture and discharge cytology are positive, but imaging modalities do not identify any cause for the discharge, surgical retromammillary cone excision may be performed.

### Assessment of Benign Solid Lesions

In K2, U2 (BI-RADS 2–3) cases, patient at normal risk (no multiple positive family history or confirmed gene mutation), with a sharp-edged, ovoid lesion not larger than 3 cm, having homogeneous structure and a longitudinal axis parallel to the skin surface, containing less than four (macro) lobulations, and displaying no hyperechoic halo sign (81–84).

• Physical examination
• Ultrasound scanning under the age of 30–35 years, complemented by mammography, if needed (suspected malignancy).
• Mammography over the age of 30–35 years, additional scans, when needed.
• Ultrasound scanning at all ages.
• Sampling: not recommended under the age of 25 years; to be considered between 25 and 30 years; strongly recommended over the age of 30 (except for unequivocal lesions such as fat necrosis, intramammary lymph node, lipoma, hamartoma).

Core biopsy is the preferred method. If, however, for any reason, cytology is performed and yields a C2 result but the report does not clearly state a definite diagnosis (e.g., fibroadenoma) the result is not acceptable. For a growing lesion, or if lesion diameter is greater than 3 cm, a core biopsy is recommended.

• If an increase in diameter of more than 20% is observed within 6 months, a core biopsy is mandatory and surgical excision should also be considered, due to the suspicion of a phyllodes tumour (85).
• For a multifocal process, sampling is recommended from the largest and/or least regular lesion.
• At any age: if no sampling is performed, follow-up is recommended every 6 months for at least 1 year; If it does not grow during this time, there is no need for follow-up.
• Biopsy is not required for macrocalcification characteristic of fibroadenoma (popcorn calcification).
• For multifocal lesions, MRI scanning for more accurate follow-up or for surgical planning is recommended.
• Cryoablation may only be performed when there is a core biopsy report (86).

### Assessment of Solid Lesions (BI-RADS 4–5) With Malignant (R5, U5), Suspected Malignant (R4, U4) or Uncertain Appearance (R3, U3)

• Physical examination (86–88).
• If a strong suspicion of malignancy arises, mammography is mandatory at all ages (including patients aged under 30) (to assess the DCIS component, etc.), with additional images, if needed.
• Ultrasound scanning (breasts + axillae) is mandatory at all ages.
• Sampling is always mandatory. Core biopsy is the preferred method and is unavoidable if a suspicion of malignancy arises on physical examination or diagnostic imaging. If, however, cytology is performed with a C1-C2-C3 finding, the result is not acceptable for excluding malignancy, in which case a core biopsy is mandatory.
• For an ultrasound-positive axilla, sampling is mandatory (cytology or core biopsy).
• For a multifocal process, if foci are not in close proximity to each other, sampling should be performed from the two furthest foci.
• For multifocal processes, preoperative MRI scanning is recommended to assess extent, especially for DCIS-associated carcinomas and lobular carcinomas.

### Assessment of Complicated Cysts

• Physical examination (11).
• Ultrasound scanning in patients under 30–35 years of age.
• Mammography in patients aged over 30–35, with additional images and ultrasound scanning, if requested.
• Doppler examination of content (growth), possibly examination of its mobility by changing body position.
• For mobile contents (i.e., clot/dense fluid) no sampling is requested for diagnostic purposes, if the cyst otherwise has a regular shape.
• Ultrasound-guided aspiration cytology of cyst fluid, and cytology or core biopsy of the solid part.
• Assessment of growth mobility with needle during sampling.
• If cyst is emptied, it is recommended that a marker clip be placed after sampling, though this is difficult to do, as this device is not currently reimbursed.
• Note: in patients aged over 30, if only one cystic structure larger than 10 mm is visible or develops in the breasts, even if it has a regular morphology, sampling should be considered due to the possibility of medullary/mucinous carcinoma/lymphoma/metastasis.

### Assessment of Calcifications

• For the analysis of questionable calcifications seen on a mammographic image, targeted zoomed or open zoomed images are suitable; there may also be a great need for these in digital mammography or synthetic 2D images (23, 89, 90).
• No sampling is indicated in cases of non-clustered, saucer-like microcalcifications with transparent centres located in the skin or just subcutaneously, or for macrocalcifications.
• With MRI scanning, the nature of the calcification cannot be defined with complete certainty; MRI therefore does not replace biopsy, and it is usually not indicated for characterization.
• Stereotactic, vacuum-assisted biopsy (VAB) is the preferred method in most cases (43). Above a diameter of 10 mm, a 12 (14)G core biopsy may also be performed, but its effectiveness (rate of non-evaluable samples, upgrade rate in final histology) is lower compared to vacuum-assisted sampling.
• If mammography of the biopsy specimen (core specimen) does not confirm any calcification, the biopsy cannot be considered representative, and a negative result, despite the calcifications described in the histological report, is not acceptable. In such cases, no therapeutic decision can be made, and follow-up alone cannot be recommended. Sampling should be repeated (mainly by vacuum-assisted method).
• If stereotaxis is not available or is available only with considerable delay, or if calcification is associated with a palpable or solid lesion identifiable on ultrasound scanning, then an ultrasound-guided core biopsy should be performed. If on mammography calcification was confirmed within the sample (core specimen), a radiopathological correlation is present, and a negative result is acceptable. In the presence of calcifications, FNA is not a suitable procedure.
• After sampling, it is recommended that marker clips be placed to identify the biopsy site and to facilitate any subsequent preoperative marking.
• For DCIS/EIC, preoperative MRI is recommended to clarify the extent of the lesion (91).

### Assessment of Architectural Distortions

• Physical examination: radial scar/complex sclerosing lesion is almost never palpable, no skin thickening/retraction is seen.
• If mammography shows architectural distortion in at least one view, additional images are required (i.e., several aspects: targeted compression without zooming, or targeted zoomed, tomosynthesis, when possible) (23, 90).
• If it can be reliably identified by ultrasound, a core biopsy or VAB with this kind of guiding, if not identifiable, then stereotactic guidance is required.
• After sampling, it is recommended that marker clips be placed to identify the biopsy site and to facilitate any subsequent preoperative marking.
• The previously used “white/black star” mammographic morphological markers are unreliable for differentiation between a tumour and a radial scar, since there are overlaps in both directions.
• MRI may help with characterization, but it does not unequivocally establish the nature of the lesion and it, therefore, cannot replace biopsy.
• If architectural distortion is only visible on tomosynthesis, conventional (2D) stereotactic guidance is not suitable for aiming, and only 3D tomosynthesis-driven stereotaxis will be adequate for this purpose. In such cases, MRI cannot replace biopsy.
• FNA is not suitable for characterizing these lesions.
• When large distortions are encountered, MRI scanning may be recommended in all cases with negative histological results, and to assess the exact extent for cases with positive histology results.

### Assessment of Asymmetric Hyperdensities

• Physical examination, careful medical history (prior surgery, etc) (23, 90).
• Mammography with multidirectional complementary images (zoomed, tomosynthesis), if needed, followed by MRI/stereotaxis if a suspicion still remains.
• Ultrasound scanning.
• If ultrasound gives negative results, an MRI should be considered, especially if a palpable/clinical abnormality is found.
• Sampling is recommended (primarily core biopsy) for any type of circumscribed abnormality found on ultrasound.
• Sampling (core biopsy or cytology) is recommended without image-guided aiming if there is a negative ultrasound but a suspicious palpable abnormality.

### Assessment of Nipple and Areolar Wounds

• Physical examination, medical history (92).
• Ultrasound scanning for patients aged under 30–35 years, but in cases of suspected malignancy, mammography should also be performed.
• In patients aged over 30–35 years: mammography, ultrasound scanning.
• Initiation of a dermatological consultation.
• Abrasion cytology sampling, indicating or performing a surgical biopsy (punch biopsy) of the wound located at the surface of the nipple/areola.
• If calcification suggestive (even only slightly) of malignancy is seen in the breast, a stereotactic biopsy is recommended.
• If on ultrasonography, a circumscribed dilated duct or a solid structure is detected, an ultrasound-guided core biopsy or possibly cytology is recommended.
• If mammography and ultrasound scanning have negative results, and nothing abnormal is revealed on dermatological consultation, but the lesion persists for a long time, MRI examination should be considered.
• If nipple biopsy is positive for tumour, an MRI scan should be considered to evaluate the extent.

### Assessment of Suspected Inflammatory Breast Cancer

• Physical examination, medical history.
• Mammography, ultrasonography.
• If pathological axillary lymph nodes are seen, they should be sampled for cytology or core biopsy.
• In the absence of abnormal lymph nodes and of detectable masses in the breast, ultrasound-guided puncture of the dilated lymphatic vessels for cytological examination may help in establishing a diagnosis.
• An ultrasound-guided core biopsy should be performed from any suspicious circumscribed area seen on ultrasound scanning.
• MRI scanning, and targeted biopsy of any detected circumscribed lesion.

### Assessment of Abnormal Axillary Lymph Nodes

• In cases of multiple axillary adenopathy, number and size range of lymph nodes showing abnormal morphology should be stated in the radiology report.
• For a known malignant lesion in the breast, FNA may be sufficient to confirm axillary metastasis.
• If mammography and ultrasound show nothing abnormal in the breast, core biopsy of the axillary lesion is preferred.
• If biopsy raises the possibility of breast origin, an MRI scan is recommended to look for an occult tumour.

### Radiological Procedures for Malignancies/Suspected Malignancies

#### For Surgery

• Preoperative marking of non-palpable breast lesions: non-palpable breast tumours are operated after preoperative localization. The lesion should be marked with ultrasound, mammography, MRI, wire hook or radionuclide (liquid or needle [seed]) (radioguided occult lesion localization, ROLL) (93, 94) guided techniques, sometimes with dye (e.g., when filling a discharging duct). The use of MRI control is justified when a lesion can only be visualized on MRI or when its extent cannot be unequivocally defined on conventional imaging.
• Combined radionuclide preoperative labelling and sentinel lymph node labelling (SNOLL) are also increasingly commonly used techniques.
• For preoperative localization, 2-view intraoperative specimen mammography or 3D tomosynthesis or specimen ultrasound is mandatory (95). A radiological report should be prepared, containing information about the presence of the abnormal lesion, the marker clip and the marking wire, and about the radiological involvement of margins.
• Sentinel lymph node biopsy (SLNB): If no metastatic lymph node is confirmed in the axilla during preoperative assessment, the sentinel lymph node should be removed as part of staging. Sentinel lymph node(s) is/are the “first” lymph node(s) on the lymphatic drainage pathway of the tumour where lymphogenous metastasis may initially develop. It/they can most effectively be identified with a combination of ^99m^Tc nanocolloid and patent blue. If there is a palpable abnormality and mastectomy is performed, the radiopharmaceutical (marker) is administered periareolarly; the radionuclide is administered by a nuclear medicine specialist and patent blue by the surgeon. If the sentinel lymph node is tumour-free, then the other lymph nodes in the axilla are also likely to be so (96).
• Preoperative localization of extensive microcalcifications (DCIS) and radial scar is recommended primarily with hookwire(s); for other lesions the radioactive localization method is more advantageous (97, 98).
• For non-palpable lesions, radioactive or magnetic labelling seeds are forward-looking approaches that can be used for labelling of both the breast and the axilla (99, 100).

#### For Neoadjuvant/Primary Systemic Treatment

• Effectiveness of neoadjuvant therapy should be monitored using appropriate imaging studies (mammography, ultrasound, breast MRI).
• For a dense breast structure, MRI scanning is the recommended method. Breast MRI shows most accurately the extent of the residual tumour and structural and size changes following treatment.
• For a good regression (downstaging) of a breast tumour (ideally at the start of any neoadjuvant treatment), an MRI-compatible metal marker should be placed in the breast tumour under image guidance, if breast-conserving surgery is possible. This kind of preoperative localization can also be performed in cases of full regression. Surgical criterion is an intact surgical margin, the achievement of which is supported by an imaging examination—preoperative breast MRI scanning (101).
• If required, a lymph node that is considered to be metastatic can be clip marked after sampling; thus, selective removal (targeted axillary sampling [TAS]) of the lymph node in question can be performed and pathological assessment of nodal regression improved.

### Image-Guided Minimally Invasive Tumour Ablation

• A promising technique for breast cancer is focused ultrasound (FUS, HIFU), a method that can be used with both ultrasound and MRI guidance. Ablation success ranges from 20% to 100%, depending on the type of the FUS system, imaging technique, ablation protocol, and patient selection (102).
• Cryotherapy is an accepted (FDA approved) method in benign cases (for histological biopsy diagnosis of fibroadenoma) (103–105). In Hungary, it is not funded by the NEAK (National Health Insurance Fund of Hungary).
• It is also a promising alternative in selected cases of malignancy and is already a subject of studies (106). A completed phase II study confirmed successful ablation in 76% of cases (107–109).
• Based on the results so far, radiofrequency ablation can be used successfully in elderly patients for whom surgery is not feasible, except for lobular carcinoma. This is not yet a practice in Hungary (110–112).
• Diagnostic image-guided vacuum-assisted excision of B3 lesions. Percutaneous, image-guided diagnostic vacuum-assisted excision has been becoming a practice in the care of smaller B3 lesions (69). Its purpose is to remove the entire lesion without surgery, usually up to a size limit of 20 mm. It is especially suitable for papillary lesions without atypia, radial scars, FEA, AEPDT, classical lobular neoplasia and mucocellular lesions. It may be indicated by the oncology team. MRI scanning may help to preclude malignancy (113, 114).

### Therapeutic Algorithm for B3 lesions

• Lesions with uncertain malignant potential (B3 lesions) represent an extremely heterogeneous group with a 9.9–35.1% risk of developing a malignant process (115–117).
• The current protocol for the treatment of B3 lesions was discussed at international consensus conferences in Zurich in 2016 and 2018. The latest recommendation 2020 on processing B3 lesions states that a multidisciplinary (oncology) team should provide an opinion on each B3 lesion.
• The recommendations for the treatment of B3 lesions after histological diagnosis are:
  ○ follow-up (mammography and/or ultrasound scanning every 6 months or annually, depending on diagnostic imaging reports).
  ○ vacuum-assisted removal.
  ○ surgical excision.

Table 2 presents the care protocol based on the “Second International Consensus Conference on lesions of uncertain malignant potential in the breast (B3 lesions)”. Table 3 shows proposed treatments for the most common lesions under the NHS (UK) protocol.

**TABLE 2:** Care protocol for B3 lesions based on the “Second International Consensus Conference on lesions of uncertain malignant potential in the breast (B3 lesions)”.

	If diagnosed by core biopsy	If diagnosed by vacuum-assisted biopsy (VAB)
ADH	Surgical removal	Surgical excision, in some cases follow-up based on the decision of the oncology team
FEA	Lesions detectable on diagnostic imaging, VAE recommended	Follow-up if the lesion detectable on diagnostic imaging has been completely removed
LN	Surgical removal or VAE (removal of a lesion visible on ultrasound scanning)	Surgical excision or follow-up appropriate for high-risk lesions if the lesion detectable on diagnostic imaging has been completely removed
PL	VAE is recommended	Follow-up if the lesion detectable on diagnostic imaging has been completely removed
PT	Surgical removal, negative surgical margin is required for borderline and malignant PT	Follow-up if the lesion detectable on diagnostic imaging has been completely removed for a benign PT
RS	VAE or surgical removal of a lesion detectable on diagnostic imaging	Follow-up if the lesion detectable on diagnostic imaging has been completely removed

ADH, temporary diagnosis corresponding to atypical ductal hyperplasia, which can only take into account the dimension seen in the biopsy sample; FEA, flat epithelial atypia; LN, classical lobular neoplasia; PL, papillary lesion; PT, phyllodes tumour; RS, radial scar; VAE, vacuum-assisted excision.

**TABLE 3:** Management protocol for B3 lesions based on NHS (UK) protocol.

Diagnosis with core biopsy (14G) or VAB	Therapeutic recommendation
Radial scar with epithelial atypia	VAE recommended, removal of 12 × 7G tissue cylinders
Papillary lesion with epithelial atypia	Surgical excision
Mucocele-like lesions with epithelial atypia	VAE recommended, removal of 12 × 7G tissue cylinders
Cellular fibroepithelial lesion	Surgical excision

VAB, vacuum-assisted biopsy; VAE, vacuum-assisted excision.

### Screening, Diagnostics and Follow-Up of Breasts That Have Undergone Cosmetic Surgery

Before cosmetic surgery (implantation, reduction, etc.): an age-appropriate imaging study should be performed to rule out a space-occupying process.

After breast augmentation for cosmetic reasons: age-appropriate screening/diagnostic tests; the same as for the normal population: mammography (with modified technique for implants: Eklund views, if technically possible), ultrasound scanning and, if necessary, guided sampling. MRI is not required by default for implanted breasts for either screening or diagnostic purposes. The most accurate method for assessing implant integrity is breast MRI. MRI scanning is also the most suitable method when imaging of the space behind the implant is required, but this is considered only in exceptional indications. Axillary silicone lymphadenopathy can be detected reliably by ultrasound, but for the assessment of other lymphatic regions (internal mammary), MRI is the suitable method.

#### Breast Implant-Associated Anaplastic Large Cell Lymphoma

The association between ALCL and breast implants with a textured surface was first suspected in 1996, with current statistics suggesting that it may occur yearly in 0.3–1/1 million women with breast implants (118). According to the literature, it is likely that there is a rare association between breast implants and the development of anaplastic large cell lymphoma, but further data is needed. BIA-LCL may be suspected 7–10 years on average after implantation, in the presence of a unilateral, increasing fluid accumulation. In such cases, cytological, bacteriological and CD30 testing of the fluid is required, and when soft tissue lesions are also present, core biopsy and MRI scanning should be considered.

### Assessment of Male Breasts

In the event of symptoms, the male breast assessment algorithm is the same as for the female breast. If a breast cancer is present, follow-up after treatment is also the same as for the female breast. Ultrasound scanning is sufficient for instrumental examination of pubertal gynaecomastia. When examining gynaecomastia in adults over the age of 30, mammography should also be performed, complemented by sampling, in doubtful cases.

Breast screening is not required in men without symptoms. Some recommendations suggest regular mammography screening for men at high risk for breast cancer (e.g., carrying BRCA gene mutation) (119–121).

### Gestational Breast Cancer

Breast cancer revealed during pregnancy or within 1 year after delivery is called gestational breast cancer.

#### Breast Assessment in Pregnant Women

Ultrasound is the primary modality for assessing a pregnant woman’s breast complaint. If necessary (e.g., suspected tumour, DCIS/EIC component, etc.) mammography can be performed observing radiation protection guidelines. Breast MRI is more difficult due to the necessity for contrast medium, as well as the increased abdominal circumference and prone position during the scan. Generally, administration of MRI contrast medium during pregnancy is a relative contraindication, but most of the contrast media approved for use in Hungary can be applied “if the clinical status of the woman necessitates it”. There are significant differences between countries and types of contrast media, so local pharmaceutical regulations should always be followed (35).

The assessment algorithm for a lactating breast is the same as for a non-lactating breast (122).

### Coding

• For multidisciplinary cooperation, it is desirable to use the following codes in radiology reports: R (1–5), K (1–5), U (1–5). The BI-RADS (0–6) code can also be entered as an option. It should be clearly indicated whether the coding is according to RKU or BI-RADS (Table 4, 5). If the two sides are not identical, the code should be entered separately (right, left) (23).
• Standardized coding facilitates clear communication between physicians. Some countries in Europe use the same system as Hungary, but the BI-RADS (Breast Imaging Reporting and Data System) scheme is internationally known and the most widespread.
• The BI-RADS system also provides precise guidance on the content of radiology reports, providing a uniform format for:
  ○ Indication for the investigation (screening, clinical study, follow-up; history data).
  ○ Type of breast structure (see Tables 6, 7).
  ○ Description of abnormalities in the breast (solid structure, asymmetry, structural disorder, calcification, abnormalities associated with the pathological process: skin thickening, nipple retraction).
  ○ Comparison with previous investigations.
  ○ Final opinion based on BI-RADS categories 0–6.
  ○ Therapeutic recommendation.
  ○ Informing the patient and the referring physician.

**TABLE 4:** RKU coding of lesions.

1	Non-pathological (negative)
2	Benign
3	Indeterminate (uncertain benign/malignant)
4	Suspicious of malignancy
5	Clearly malignant

R, radiology = mammography; K, clinical/physical examination; U, ultrasound scanning.

**TABLE 5:** BI-RADS coding of lesions for mammography and ultrasound (MRI BI-RADS differs from this).

0	Incomplete assessment: additional imaging investigation(s), or comparison with previous ones is/are required
1	Negative
2	Benign
3	Probably benign: short-term (6 months) follow-up or biopsy required (probability of malignancy below 2%)—screening cannot be coded directly as 3
4	Suspected malignancy: histological diagnosis (core biopsy) required (probability of malignancy between 2% and 95%)
4a	Low probability of malignancy (2–10%)
4b	Intermediate probability of malignancy (10–50%)
4c	High probability of malignancy (50–95%)
5	Most likely malignant (≥95%): histological diagnosis required
6	Malignancy confirmed by biopsy: appropriate management is required

**TABLE 6:** BI-RADS classification of breast structure types.

BI-RADS A	The breast is almost entirely adipose in structure, the sensitivity of mammography is high
BI-RADS B	Scattered glandular areas of fibroglandular structure
BI-RADS C	Heterogeneously dense glandular parenchyma, that may mask minor lesions
BI-RADS D	Markedly dense glandular parenchyma, the sensitivity of mammography is low

**TABLE 7:** Breast structure types according to Tabár.

Glandular	T1
Adipose	T2
Fibroadipose	T3
Adenotic	T4
Fibrotic	T5

### Interdisciplinary Cooperation

• Sample handling, cooperation between radiology and pathology.

Aspiration cytology sampling should be performed using a syringe with a rubber stopper. The radiologist performing the sampling should consult the evaluating cytopathologist about the method of smear preparation and fixation, considering that the type of staining used for smear evaluation determines method of fixation, and inadequate smearing may lead to a non-evaluable sample.

• The test order attached to biopsy specimens (preferably a complex radiology report) should include the radiologist’s opinion, as well as relevant clinical data available to the radiologist (e.g., any other tumour disease the patient may have).
• Summary report and breast/oncology team opinion:

After each biopsy, regardless of whether the radiological/pathological/clinical opinions are consistent or contradictory, a written diagnostic “Summary Report” must be prepared. This will be issued by the radiologist performing the biopsy and summarizing tests (after consulting with the pathologist, in questionable cases). The purpose of the diagnostic “Summary Report” is to synthesize the results of different (radiological and pathological) diagnostic methods to facilitate further action and/or a therapeutic decision. Based on the results of the assessment, the breast oncology team gives a therapeutic recommendation, possibly proposing complementary tests; all these are recorded in writing in the “Opinion of the Breast Oncology Team”.

## Investigation Methods for Staging and Monitoring of Breast Carcinoma (Other Than Breast Tests)

### Methods for Investigating Regional Lymph Nodes

• Ultrasound scanning (35, 65, 66).
• Radionuclide lymphoscintigraphy (radionuclide localization of sentinel lymph nodes) (CT, MRI, PET/CT).

### Methods for Investigating Location of Distant Metastases

• Chest, lungs: chest X-ray, CT.
• Mediastinum: CT, MRI, PET/CT (whole body information).
• Chest wall: CT + US, MRI.
• Abdomen: US CT, MRI, PET/CT (whole body information).
• Bone: scintigraphy, 18F-NaF PET (-based measurements) (not yet funded in Hungary), conventional X-ray, MRI, CT, 18F-FDG PET/CT (whole body information) (35, 38).
• Central nervous system:
  ○ brain: MRI, CT.
  ○ spinal cord: MRI.
• Lymph nodes (non-regional): US, CT, MRI, 18F-FDGPET/CT (whole body information).

## Methods for Assessing and Monitoring the Pre- and Post-Treatment Stage

The stage of the disease is determined based on tumour size and certain specific features, regional lymph node involvement, and the absence or presence of distant metastases (35, 36, 38, 123–125).

### For *In Situ* (Stage 0) and Early Invasive (Stage I, II) Breast Cancers

#### Staging

Of the regional lymph nodes, assessment of the axilla is a mandatory part of the ultrasound scanning of breasts, complemented by guided sampling in the event of any suspicion. No other imaging tests for staging are required if the case is detected by screening, is stage T1N0, has a favourable histology result.

(Note: baseline imaging studies may only have the benefit of providing a basis for comparison for subsequent radiological examinations performed for any reason, such as recording the size and morphology of benign lesions). This may later spare the patient from technically difficult, burdensome biopsies and may make follow-up examinations unnecessary. 18F-FDG PET/CT in the early stages is only recommended for N2–3. At an early stage (I, II, operable III), it may be justified if other investigations or clinical conditions suggest distant metastasis.

### For Stage III, IV Breast Cancer and Biologically Aggressive Tumours

#### Staging

Regions of the neck, chest, abdomen, lesser pelvis:

• CT scan: With MDCT (multi-detector, multislice CT).
• PET/CT is recommended in all cases (stage IIB–IV) when the risk of distant metastases is high; it has been shown to perform better than diagnostic CT staging, and for cases with uncertain or inconsistent results obtained using other procedures. Inspiratory chest CT should also be performed during the PET/CT scanning, if not already performed. If the result of PET is not conclusive for clarifying liver lesions, liver MRI is warranted. If FDG-negative sclerotic bone lesions suggestive of metastases are visualized on PET/CT, bone scintigraphy with SPECT/CT measurements is required. Bone scintigraphy can be replaced by 18F-NaF PET/CT (currently not funded in Hungary).

#### Follow-Up of Treated Breast Cancer Patients

• Mammography + ultrasound scanning of the treated breast every year for 5 years (unless otherwise specified by the oncology protocol relevant for the patient). After that, annual mammography is recommended.
• Similar actions are required after reconstructive breast surgery, if no implant was used for reconstruction.
• For a breast reconstructed with an implant, modified mammography (Eklund views) + ultrasound should be performed. By default, MRI is not required for implanted breasts for either screening or diagnostic purposes.
• A complex assessment of the contralateral breast is performed annually.
• Even after mastectomy, mammography can almost always be performed on the remaining tissue.
• Breast MRI is indicated after prior consultation with a radiologist:
  ○ in highrisk cases (young patient, dense breast structure, genetic, familial risk).
  ○ if recurrence cannot be confirmed by conventional radiological imaging, though it is suspected based on the clinical picture.
  ○ in other difficult and contradictory cases.
  ○ due to limited evaluability, MRI is generally not recommended for 6 months after surgery and within 12–18 months after radiation therapy, except for special cases.
• Other imaging tests (e.g., PET/CT) are recommended only if a clinical suspicion arises, being complemented with image-guided sampling, if needed.
• In case of confirmed recurrence, core biopsy is definitely recommended for the assessment of histological parameters.
• Adequate laboratory and imaging tests are recommended to monitor the side-effects of therapy, according to the protocol.
• PET/MRI is currently only available in clinical trials and is currently not funded.

#### Monitoring of Therapeutic Response Using Radiological Examinations

If there is known dissemination, the oncologist or the treatment protocol will determine the time of follow-up. The choice of imaging method is a joint decision of the attending physician and the radiologist, taking into account the possibility of visualization, availability, and reimbursement (9, 126, 127).

### Nuclear Medicine Investigation Methods for Staging

Bone scintigraphy: a nuclear medicine method based on a radionuclide technique. Planar whole-body scanning is considered to be the standard procedure. Currently, bone scintigraphy may be complemented by single-photon emission tomography (SPECT) or hybrid SPECT/CT measurements, in order to increase diagnostic accuracy (37, 94).

^99m^Tc phosphonate analogues used for scintigraphy show good bone binding and are rapidly washed out from soft tissues. The sensitivity of the test is 90–100% and specificity is around 50–60%. Increased radiopharmaceutical accumulation can be seen in abnormal, metastatic areas due to increased osteoblast activity and enhanced blood perfusion. Bone scintigraphy usually shows lesions significantly earlier than conventional radiological methods. Due to the method’s relatively low specificity, 18F-NaF PET/CT (bone PET) is increasingly used in countries that are well-equipped with PET systems (35).

PET and SPECT (hybrid forms of PET/CT and SPECT/CT): The essence of these nuclear medicine techniques is that they map the temporal and spatial distribution of selected pharmaceuticals, molecules, drugs, etc. (biomarkers, radiopharmaceuticals, radioligands, tracers, etc.) labelled with PET or SPECT isotopes. Photons emitted from the patient are detected in three dimensions (3D) and quantified, or measured semi-quantitatively. Therefore, in addition to the technical development of these systems, use of various tracers and biomarkers is one of their theoretically unlimited strengths. Incorporation of PET and SPECT cameras and radiological imaging equipment (CT, MRI) into a single machine (PET/CT, PET/MRI, SPECT/CT) has significantly decreased examination time (whole body imaging takes 6–10 min) and amount of radioactivity, as well as enabling simultaneous data collection, accurate measurement and localization of quantitative data of functional molecular maps. As a result, diagnostic accuracy and reliability have significantly improved. As well as increasing the high sensitivity, specificity, and positive and negative predictive value (PPV and NPV) of PET and SPECT tests, the use of hybrid techniques also proved to save time and money and allowed the use of significantly lower activities.

Whole-body-18F-FDG PET/CT: provides whole-body information in a single session at a lower radiation exposure than standard contrast-enhanced CT scan(s), identifies distant metastases with the highest sensitivity, and may help to detect possible second primary tumour(s). During evaluation of post-therapeutic lesions and identification of recurrences, as well as being a highly sensitive method, the extent of the disease and possible progression can be visualized using a lower radiation exposure and in a time-saving manner.

18F-NaF-PET/CT: also called “bone PET” may be chosen as an alternative to bone scintigraphy (29–35). In M-staging, a combined use of 18F-FDG and 18F-NaF tracers provides the highest sensitivity, specificity and diagnostic accuracy.

PET/Magnetic Resonance Imaging (PET/MRI): currently this is primarily used in research (37, 40, 41).

#### Use of Whole-Body Bone Scintigraphy, Complemented With SPECT/(CT), If Needed

Whole-body bone scintigraphy is recommended at an early stage, where the clinical risk of bone metastasis is high at the time of diagnosis and in patients with stage III or IV breast cancer at the time of diagnosis, even in asymptomatic and complaint-free patients (31, 32). Examination is also justified if there is clinical, laboratory or radiological suspicion of bone metastases, during follow-up and long-term care of patients.

For lesions that are unequivocal on bone scintigraphy, it is recommended to complement the scintigraphy with a SPECT, preferably SPECT/CT test to improve diagnostic reliability of bone scintigraphy. SPECT/CT is also recommended for solitary metastases, e.g. when vertebral metastases are suspected, in order to differentiate degenerative and metastatic processes.

#### Use of 18F-FDG PET/CT

This method is an important step in staging and re-staging assessments, in the event of suspected recurrence, and in all cases where an issue cannot be judged properly using conventional imaging studies or if clinical and imaging data are contradictory or uncertain. The main indication for PET/CT is the assessment of equivocal or suspicious lesions in cases at high risk for metastasis or of already known metastatic disease (35). In view of the whole-body information provided by 18F-FDG PET/CT, this test may be more beneficial than routinely used conventional staging methods in terms of reduced time, costs, and radiation exposure.

For *in situ* and low-risk early (stage I-II) breast cancers, 18F-FDG PET/CT is not recommended as a routine method since:

• It cannot replace sentinel lymph node biopsy.
• In the detection of small metastatic lesions, below the resolution limit of the equipment (typically <5 mm in diameter), the sensitivity of PET/CT is low.

The use of 18F-FDG PET/CT is recommended for:

• Breast cancers that are early stage (I, II) according to conventional staging, but are at high risk for metastases.
• Stage III and IV patients.
• The assessment of recurrences to evaluate the extent of the process, especially for distant metastases (35, 38).
• Differential diagnosis of brachial plexopathy, differentiation between a viable tumour and necrosis/scar tissue, when this is of crucial importance.
• The evaluation of parasternal or mediastinal lymph node metastases—with adequate FDG avidity (IDC-NST, Ki67 > 20%), when PET/CT performs better than other imaging methods.

#### The Role of PET/CT in the Detection of Bone Metastases

• Bone scintigraphy is more sensitive for osteoplastic metastases, while 18F-FDG PET/CT is more sensitive for lytic and mixed metastases. The two methods do well to complement each other (29–35, 65, 66, 94).
• For screening of bone metastases, bone scintigraphy continues to be the method of choice, complemented by SPECT or SPECT/CT, if needed.
• If bone scintigraphy is negative or uncertain and if there is a strong clinical suspicion of bone metastasis, 18F-FDG PET/CT scanning is recommended (for the assessment of lytic and mixed metastases).
• If 18F-FDG PET/CT has been performed in a patient for any reason and bone metastases have been confirmed (consistently in PET and CT modalities), bone scintigraphy is not required (35).
• If the patient has had FDG PET/CT and on CT scan a sclerotic lesion suggestive of metastasis has been visualized, which though FDG-negative may be a viable bone metastasis, bone scintigraphy with SPECT or SPECT/CT measurements is recommended to confirm this.
• 18F-NaF PET/CT is a method used as an alternative to bone scintigraphy (a procedure that is not yet reimbursed in Hungary). Also known as “bone PET”, it detects skeletal changes with the highest sensitivity (35).

## Report Templates and Communication

• Standard report coding and the use of common templates make written reporting (which represents a significant part of radiology work) more accurate and easier, facilitating a closer relationship between radiologist and clinician, effective communication between disciplines and the development of a common language. For development of a common reporting nomenclature, the introduction and consistent use of BI-RADS atlas terms in breast testing is extremely important (23, 128, 129). However, the development of a specific report format is the prerogative of each institution. The standard basic report templates are, on the one hand, recommendations for the format of negative reports (mammography, ultrasound, breast MRI), and, on the other hand, special morphological descriptions of certain pathological lesions. Based on templates, selecting the appropriate option, custom reports may be created, including any specific content when needed.
• The first step in the timely detection of cancers is to provide accurate and comprehensible information to patients about the radiological examinations that are recommended according to patient age, device availability and indications. In addition, efforts should also be made to familiarize patients as much as possible with the predisposing factors for breast cancer, prevention options and risk factors, and the importance of breast density should also be emphasized. Fortunately, there are increasing numbers of more effective campaigns, and more non-profit organizations are undertaking awareness-raising activities. The internet and various social media platforms are also good opportunities for providing information.
• In everyday practice, in addition to the importance of detailed information prior to examinations (informed consent forms), the focus should also be on proper (in-person) communication of investigation results (histological reports, plans for further action, etc.). Trust and collaboration are not only cornerstones of effective doctor-patient communication, but in some cases are also the cornerstones of healing. Breast diagnostics is an area of radiology in which this is of crucial importance.
• With the introduction of the EESZT (Electronic Health Service Space), patients also have access to interim results from pending assessments. This may lead to misunderstandings of diagnoses, inappropriate, self-initiated modification of patient pathways, and overload of the health care system.

In situations where a decision (e.g., therapy or ending the assessment process) is made based on a common end result of related reports, it is appropriate to make a definite reference to this at the end of each report. For example: “We will offer a “summary opinion” based on the pathology report of the targeted sampling performed today together with the radiology report. We ask the patient’s attending physician to wait for the ‘summary report’ when deciding on the therapy, since its content will not necessarily be the same as the content of the two separate reports!”.

## Competences, Legal and Verification Issues

### Professional Staff

• According to the professional recommendations of the Breast Diagnostic Section of the Hungarian Society of Radiologists, breast imaging tests and image-guided breast interventions may only be performed by a radiologist who has passed the “Complex Radiological Breast Diagnostics” licensure exam (130), with the required minimum technical conditions.
• According to the current requirements of the Minimum Conditions Act (131): at least one licensed specialist must work in a workplace.
• MRI scanning of the breast is also subject to the provisions of licensure exams for breast diagnostics, so breast MRI reports must be produced by a radiologist with such a qualification (or jointly with a licensed radiologist).
• Mammography may be performed by a medical technician with a specific qualification (X-ray technician, radiographer, diagnostic and interventional imaging technician, diagnostic imaging technician).
• The competences of a sonographer do not include the evaluation of breast ultrasound at any age or indication.
• Nuclear medicine investigations: nuclear medicine specialist, specially trained technician.
• Reports of hybrid examinations (PET/CT or PET/MRI) should be compiled jointly by a nuclear medicine specialist and radiology specialist with appropriate experience.

### Issues Regarding Forensic Experts

#### Disputed Radiological Services

In the event of a dispute (e.g., an action for damages), it is up to an expert with proven experience in mammography screening and diagnostics to consider whether the service was provided based on the principle of utmost care. The opinion of a non-radiologist, a general radiology specialist, or a radiologist working occasionally in a low-throughput mammography workplace may not be accepted as an expert opinion. Only the opinion of a radiologist who has passed a complex radiological breast diagnostic licensure test and who has proven to be highly experienced in the given area (e.g., screening, breast MRI) may be decisive.

In order to give an opinion, the expert should simulate a real-life situation; they should not analyse the appropriateness of preoperative diagnosis and therapeutic decision retrospectively, with the benefit of detailed results of all investigations and surgical and histological reports, but it is recommended that they form an opinion only on the basis of the information that was available at the time of the decision(s) contested in the lawsuit.

#### Disputed Complex Care

Since decision-making about breast diagnostics and therapy requires the synthesis of many aspects (according to the protocol, it is a multidisciplinary (team) activity), it is recommended that forensic expert opinion be reached in a similar way, by a team with appropriate experience, as is the practice in some developed countries. It is not acceptable for a complex process to be evaluated by the representative of only one of the disciplines.

#### Penalties

• Since the inadequate performance of screening or diagnostic units may jeopardize the lives of many women, greater emphasis should be placed on licensing, quality assurance, and regular supervision of licensees.
• Regular inspections of workplaces performing breast screening and diagnostics are essential, looking at operating conditions, minimum professional (personal and material) conditions and radiation protection.
• The content of the contract signed when opening a screening centre should be verified, and if any errors are revealed, the screening centre may be excluded or replaced with other suitable centres.
• In the event of improper functioning, it is recommended for both screening and diagnostic centres that a warning and an appropriate deadline for correction be given, and that if this deadline is not met, the licence of the centre should be revoked. In the event of a serious fault or deficiency, operation must be discontinued immediately.

#### Interval Cancers

Mammography screening is an effective but not perfect method: among the group of people receiving a negative result, development of some new cases of cancer in the subsequent screening interval is inevitable. However, the incidence of interval cancers should be kept to a minimum, and their number should be recorded centrally and closely monitored (132).

The history of interval cancers should be systematically traced (in a well-functioning, accessible, searchable national registry).

## Recommendations for Further Development of Hungarian Breast Cancer Screening and Diagnostics

Screening of high-risk women (133): Within and in parallel with public health screening, high-risk groups should be identified and separated, and these groups should be screened according to a separate protocol, their data should be collected separately, and separate information materials should be compiled for them. This necessarily requires the expansion of breast MRI and MRI-guided sampling capacity and extensive training of professionals, as well as the collaboration and training of geneticists.

Screening for the 40–44 age group: The known lower performance of mammography for young people is explained by lower parenchyma density, and, due to a lower incidence of breast cancer, decrease in mortality is also lower. At this age, however, tumours may be significantly more aggressive (50).

Recommendation: the professional and financial implications of screening in the 40–44 age group should be examined, and the screening age should be extended accordingly.

Screening of older women: It is recommended that screening be continued over the age of 65 if there is no other serious illness that worsens life expectancy (expected to result in death within 3–5 years). Carcinomas in women aged 65 years or over account for 45% of all new breast cancers, and 45% of deaths from breast cancer also occur in this age group.

Recommendation: professional and financial implications of screening in the 66–75 age group should be examined, and the upper age limit for screening should be extended, in accordance with European practice. When the upper age limit for organized screening is reached, it is recommended that everyone is automatically sent an information letter with an offer to continue screening individually.

Partial increase of screening frequency: According to several international resolutions proposed over recent years, the recommended screening interval for all ages is 1 year. This is due to a lower sojourn time at a younger age, resulting in a significantly higher rate of interval cancers. We also refer here to the practice of Sweden (18 months between age 40–45 and 24 months between age 45–75) and of the United States (12 months), which have the longest screening experience. As the incidence of interval cancer is higher, especially at a younger age, introduction of a screening interval of 18 months is recommended, especially in the of 40–54 age group (50, 132).

Recommendation: examine the professional and financial implications of more frequent screening in this age group and introduce it accordingly.

Digital mammography: In Hungary, the analogue-to-digital switchover has taken place for the majority of mammography devices, at all official screening stations, and this needs to be completed. Since the primary goal of breast screening is to reduce mortality due to breast cancer, this goal can be achieved when the tumour is diagnosed in its initial stage or in a precancer state, which requires optimal technical conditions. Analogue (X-ray film) technology has been excluded from breast diagnostics worldwide and has been replaced by direct digital technology, since direct digital technology has a significantly higher sensitivity for the detection of early breast cancer and DCIS than analogue X-ray film technology. According to the literature, the direct digital technique has revealed twice the number of DCISs, including 8% more high-grade DCISs, than conventional X-ray film mammography (134). For dense breasts, difference between the sensitivity of these two techniques is particularly great in favour of digital technology, and this is of importance primarily for pre- and perimenopausal women and for those under the age of 50. Another important aspect is that direct digital technology uses a lower radiation dose for the patient. Other advantages include fast imaging, possibility of post-processing, easier image storage and reproducibility, and the possibility of telemedicine. When direct digital mammography is used, compliance with technical requirements and quality control are prerequisites for the applicability of the method in breast screening (3, 135–141).

The use of phosphor storage plate digital technology (CR) in breast screening and diagnosis is strongly contraindicated, since its spatial resolution is lower than that of mammography film and direct digital technology, it requires a higher radiation dose, and some microcalcifications (low-density “powder” calcifications) may remain undetected (142).

Tomosynthesis (3D mammography with 2D synthetic software) is not yet a definite recommendation in international screening protocols, but its use is already clearly recommended in screening and diagnostic algorithms for certain cases (e.g., high-risk women). Research is ongoing into use of this method for screening women at normal risk, with promising results. Reducing the number of interval carcinomas is the issue currently being studied, before the method may be introduced more widely. This method may already be used in individual screening, and it is not contraindicated in organized screening. We recommend unconditional support for tomosynthesis and purchasing new mammography units equipped with this option.

Artificial intelligence: it is recommended that this topic be closely monitored. Gradual introduction in screening should be considered if scientific evidence emerges. At present, none of the dual-reading radiologists can in any way be replaced by AI, since the scientific evidence is not yet sufficient.

Stereotaxis and vacuum-assisted biopsy: These methods have been the gold standard in international practice for many years for the diagnosis of lesions (primarily microcalcifications) that can only be seen on mammography (47). Recommendation: We recommend promoting wider use of these methods in Hungary by settling reimbursement, removing the reimbursement constraint (EFI), and allocating much larger capacity, i.e., a higher number of eligible investigation sites.

BI-RADS: A switch to the more widely used BI-RADS radiology coding of the American College of Radiology (ACR) is recommended, since this is more appropriate than the RKU coding currently in use and also more in line with pathological coding. This system has been continuously updated since 1993, and is optimized to support diagnostic and therapeutic strategy, quality assurance, audit, and better data collection (23).

Renewal of radiation protection regulations and inspection procedures to reduce radiation exposure among the population. Development and integration of quality assurance in digital mammography (125, 143).

Limiting the scope of the license exam to persons: It is proposed to amend the Minimum Conditions Act to make the licensing examination mandatory for each radiologist performing breast examinations, individually. According to the present regulation one single person with such a qualification is sufficient in a workplace, which is not safe enough. More inspections are recommended, and authorizations should be revoked if necessary.

Reimbursement: Reimbursement of breast screening and diagnostics has not changed for more than 15 years or it is not reimbursed, despite the spread of advanced techniques (e.g., digital mammography, tomosynthesis, marker clip, 18F-Na-F PET/CT, tomo-guided stereotaxis, etc.). Some procedure codes are completely missing from the list of activities that are publicly funded (by the National Health Insurance Fund of Hungary, NEAK) or are unduly restricted (e.g., individual funding for vacuum biopsy, performance-volume limit for diagnostic/therapeutic care of those recalled from screening, one FNA/core biopsy limit for one appearance, etc.). All the above hinder performance and development, and ultimately the entire modern patient care, and they should be reviewed. It is recommended that the Secretary of State for Health take steps to arrange for adequate funding for screening and diagnostic procedures and to review this automatically at least every 2 years.

### Organizational Proposals for Screening

Objective: to update the Hungarian screening model in every direction, to meet European standards and to maximize efficiency (144, 145).

Interdisciplinary National Screening Working Committee: in 2001, the task of the Interdisciplinary Working Committee, set up by the National Chief Medical Officer, was to give an opinion on the reports of screening centres, to participate in the periodic on-site inspection of centres and in inspections prior to licensing. The procedures of the Working Committee need to be renewed, it needs to operate continuously, and it needs to be granted stronger rights. Otherwise, the existence of personal and financial conditions of screening can be assessed only to a limited extent, its effectiveness can only be estimated, without statistical evidence, and the effectiveness of screening may diminish.

We recommend a reasonable adaptation of screening centres (a reduction in numbers, strict quality control), compliance with technical and radiation protection requirements, and support at organization level (regular condition and stability tests, dosimetry).

It is considered necessary to systematically monitorize the collection of statistical data (e.g., registration of prevalence/incidence screening cycles), to assess the effects of screening based on state-of-the-art breast cancer mortality statistics, to ensure follow-up of patients who have been recommended for surgery but who are lost to follow-up (approx. 34%) by possible merger of the EESZT (Electronic Health Service Space) system.

It is considered important to develop a strategy that is effective at increasing participation (reducing the disadvantages of selection bias), maintaining and verifying regular appearance, with the involvement of trained screening statisticians. This is based on rationally designed screening plans (coordinated activity of the screening centre and entity organizing the screening), specification of call lists, monitoring and correction of professional updates and changes.

Rationalization of calls to the same location and time (individual attendance at the same time of the screening interval ±1 month). Consideration of territorial characteristics, seasons, seasonal occupancy aspects, consultation with the creators of lists.

Arranging door-to-door transport via screening coordinators, using funded services of the local bus company. Expected benefit: more comfortable, cheaper travel, better attendance rate.

For conditions and expected results, see EU breast screening indicators and the earlier Hungarian screening-diagnostic protocol (1, 2, 90, 146–148). These previous materials are only partially up to date, and require constant updating by the Interdisciplinary Working Committee.

## Breast Self-Examination Is Not a Screening Test

There is evidence that self-examination does not reduce mortality, therefore it should not be suggested that by carrying out self-examination women are substantially benefiting their health or acting against breast cancer (IARC 2015, ACS 2015 recommendations). It should be also noted that medical physical examination does not improve mortality rates either. The state-of-the-art message is: “Women of screening age should have regular mammography screening!” (149).

## Media Communication and Protection Against Attacks on Breast Screening Mammography, and Public Advertising and Use of Non-Evidence-Based Methods

Attacks on breast screening mammography, which has a 40-year history and is backed by strong evidence, and public advertising and use of non-evidence-based, pseudo-scientific methods: these endanger women and decrease the trust in the medical profession and in our achievements so far. These new trends, which lack any foundation, irresponsibly offer “more effective” diagnostic (and therapeutic) methods in the place of the methods and tools used in academic medicine for cancer screening, with an overemphasis on the disadvantages of these methods (e.g., radiation exposure, breast compression). Although these so-called “alternative” test methods detect hardly any (or only a very limited number of) possible lesions in the breast, women still opt for them because they promise to be simpler and offer less discomfort. They are not aware that this deception, which is lacking in any scientific basis, may cost them their lives.

We are observing with great concern how these “testing methods”, which do not meet the criteria of evidence-based medicine, have not been evaluated in appropriate clinical trials and do not comply with professional rules of medicine or internationally accepted principles, are advertised without any hindrance by their service providers, and even though these providers are not licensed for such activities, the authorities have not taken effective actions against them. The Radiology Section of the National Advisory Board, the Breast Diagnostics Section of the Hungarian Society of Radiologists and the Hungarian Cancer League have already acted against these “diagnostic methods” and against the advertisement of medical diagnostic methods, but so far with no result.

As physicians, it is our moral duty to raise our voices very strongly to protect women. Therefore, we hereby repeatedly and strongly urge the competent ministry to be partners in eradicating this unsustainable situation.

Our recommendations:

• The Secretary of State for Health should: take a stand on the issue and communicate this to professional organizations.
• Enable the public to be informed about the serious dangers of these activities in the public service media through awareness-raising public service advertisements, similar to traffic safety ads.
• Employ a press and advertising professional to develop a strategy, working with physicians, to eradicate once and for all this phenomenon which threatens women’s lives.
• Submit a bill to parliament making it illegal to conduct or promote pseudo-scientific medical activities.

This is part 2 of a series of 6 publications on the 1st Central-Eastern European Professional Consensus Statements on Breast Cancer covering imaging diagnosis and screening (present paper), pathological diagnosis (150), surgical treatment (151), systemic treatment (152), radiotherapy (153) of the disease and related follow-up, rehabilitation and psycho-oncological issues (154).

## Author’s Note

The consensus document contains product placement without the intention of advertising. Each complex molecular test is unique, and although these can be described without indicating their name (for example with the number of genes tested), not everyone will necessarily understand what this refers to. For this reason, and adopting the practice used in some of the source works, the tests are listed under their trade name.
